# DectiSomes: Glycan Targeting of Liposomal Drugs Improves the Treatment of Disseminated Candidiasis

**DOI:** 10.1128/AAC.01467-21

**Published:** 2022-01-18

**Authors:** Suresh Ambati, Tuyetnhu Pham, Zachary A. Lewis, Xiaorong Lin, Richard B. Meagher

**Affiliations:** a Department of Genetics, University of Georgia, Athens, Georgia, USA; b Department of Microbiology, University of Georgia, Athens, Georgia, USA

**Keywords:** *Candida*, candidiasis, targeted delivery of antifungals, Dectin, DectiSomes, amphotericin B, anidulafungin, AmBisome, *Candida albicans*

## Abstract

Candida albicans causes life-threatening disseminated candidiasis. Individuals at greatest risk have weakened immune systems. An outer cell wall, exopolysaccharide matrix, and biofilm rich in oligoglucans and oligomannans help *Candida* spp. evade host defenses. Even after antifungal treatment, the 1-year mortality rate exceeds 25%. Undoubtedly, there is room to improve drug performance. The mammalian C-type lectin pathogen receptors Dectin-1 and Dectin-2 bind to fungal oligoglucans and oligomannans, respectively. We previously coated amphotericin B-loaded liposomes, AmB-LLs, pegylated analogs of AmBisome, with the ligand binding domains of these two Dectins. DectiSomes, DEC1-AmB-LLs and DEC2-AmB-LLs, showed two distinct patterns of binding to the exopolysaccharide matrix surrounding C. albicans hyphae grown *in vitro*. Here we showed that DectiSomes were preferentially associated with fungal colonies in the kidneys. In a neutropenic mouse model of candidiasis, DEC1-AmB-LLs and DEC2-AmB-LLs delivering only one dose of 0.2 mg/kg AmB reduced the kidney fungal burden several fold relative to AmB-LLs. DEC1-AmB-LLs and DEC2-AmB-LLs increased the percent of surviving mice 2.5-fold and 8.3-fold, respectively, relative to AmB-LLs. Dectin-2 targeting of anidulafungin loaded liposomes, DEC2-AFG-LLs, and of commercial AmBisome, DEC2-AmBisome, reduced fungal burden in the kidneys several fold over their untargeted counterparts. The data herein suggest that targeting of a variety of antifungal drugs to fungal glycans may achieve lower safer effective doses and improve drug efficacy against a variety of invasive fungal infections.

## INTRODUCTION

Invasive candidiasis is among the top four most life-threatening fungal diseases (1–5). Most *Candida species* that cause disseminated candidiasis such as Candida albicans and Candida glabrata are commensals found in the gastrointestinal and urinary tracts and rarely cause invasive infections in healthy people. However, immunocompromised individuals such as patients on immunosuppressants as part of cancer treatment or cell or organ transplant therapy are particularly susceptible (6–8). Candidiasis is the most common invasive fungal disease of HIV patients who developed AIDS (9, 10). Even with antifungal drug therapy, the 1-year mortality rate with disseminated candidiasis ranges from 25% to 40%, depending upon the patient’s underlying conditions (3, 5, 9, 11–14). When *Candida* infections spread to the central nervous system and brain, the mortality rate approaches 90% (15). The annual medical costs from disseminated *Candida* spp. infections in the United States were recently estimated at 3 billion dollars, a third of the cost to treat all fungal diseases, and representing 45% of the U.S. hospitalizations from fungal infections (4, 16). Per patient treatment costs for candidiasis range from $40,000 to $150,000 (3, 5, 16–18). Clearly, there is a considerable need for improved antifungal drug performance.

Recommended antifungals to treat invasive candidiasis include the polyenes (e.g., amphotericin B, AmB), echinocandins (e.g., anidulafungin, AFG), and azoles (e.g., fluconazole) (19–22). Resistance to various antifungal drugs is a serious emerging problem (23–25). AmB was the first to be used to treat invasive candidiasis, but at effective doses and with extended treatment times, AmB and other polyenes cause renal toxicity (26–28). Because of its nephrotoxicity, AmB has been replaced by echinocandins such as AFG as the first line clinical treatment (19, 22). Lowering the effective dose, while improving the efficacy of various antifungal drugs would dramatically expand our treatment options for candidiasis (19, 29, 30).

AmB is amphiphobic and quite insoluble in aqueous solutions; therefore, clinical formulations often include AmB loaded into the nonpolar interior of detergent micelles (e.g., AmB-DOC) or intercalated into the bilipid membrane of liposomes (e.g., unpegylated liposomal AmB, AmBisome, L-AmB) or our pegylated liposomal version AmB-LLs (31, 32). Current antifungal preparations used in the clinic have the disadvantage that they deliver drug to fungal and host cells alike, and have little specificity for fungal cells. We define DectiSomes as liposomes coated with a protein that targets them to a pathogenic cell, thereby increasing drug concentrations in the vicinity of the pathogen and away from host cells (33). We previously made two classes of DectiSomes, DEC1-AmB-LLs and DEC2-AmB-LLs, by coating AmB-LLs with the carbohydrate recognition domains of Dectin-1 (32) or Dectin-2 (31). Dectin-1 (*CLEC7A*) and Dectin-2 (*CLEC4N*) are human pathogen receptors expressed on the surface of various leukocytes that recognize fungal beta-glucan and alpha-mannan containing oligosaccharides, respectively. Both glycans, the ligands for targeting by these two classes of DectiSomes, are expressed in cell walls, glycoproteins, exopolysaccharide matrices, and/or biofilms of most pathogenic fungi, including *Candida* spp. (34). *In vitro* studies show that relative to untargeted AmB-LLs, DEC2-AmB-LLs bind to different developmental stages of *C. ablicans*, bind 100-fold more strongly, and bind primarily to oligomannans in their extracellular matrix. Furthermore, DEC2-AmB-LLs kill or inhibit the growth of *Candida* cells one to two order(s) of magnitude more effectively than AmB-LLs (31). Recently, we showed that DEC2-AmB-LLs were significantly more effective at reducing fungal burden of Aspergillus fumigatus in the lungs and improving mouse survival than AmB-LLs in a neutropenic mouse model of pulmonary aspergillosis (35). Herein, we examine the efficacy of these same DectiSomes to control C. albicans in a neutropenic mouse model of disseminated candidiasis. In addition, we included the preparations and initial examinations of AFG loaded DectiSomes and a Dectin-targeted AmBisome.

## RESULTS

### DectiSomes bind efficiently to *in vitro* grown C. albicans hyphae.

**FIG 1:**
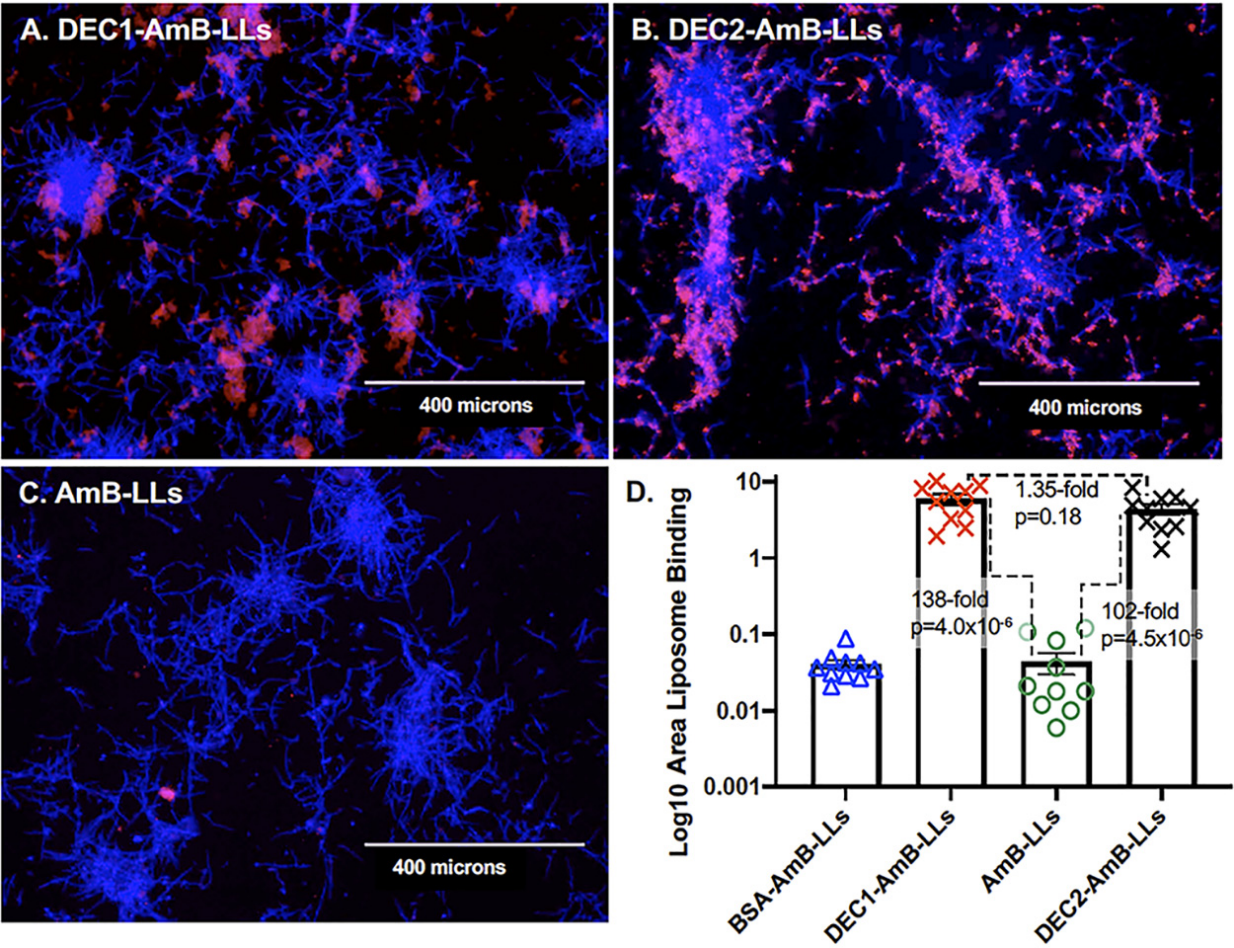
DEC1-AmB-LLs and DEC2-AmB-LLs bound specifically to *in vitro* grown C. albicans hyphae. C. albicans hyphae were stained with rhodamine tagged liposomes (A) DEC1-AmB-LLs and (B) DEC2-AmB-LLs, and (C) AmB-LLs. An image of BSA-AmB-LL binding is not shown. (A to C) Fungal cell chitin was stained with CW. The epifluorescence of chitin (blue) and liposomes (red) was photographed at ×10 magnification. All four preparations of liposomes were diluted equivalently. Size bars represent 400 microns. (D) A scatter bar plot compares the area of red fluorescent staining quantified from 10 images for each type of liposome. Standard errors and the fold differences in average area of staining and *P* values are indicated for comparisons of DectiSomes to AmB-LLs.

The binding of DEC1-AmB-LLs to C. albicans has not been studied in as much detail (32) as DEC2-AmB-LL binding (31). The binding of rhodamine A tagged DEC1-AmB-LLs and DEC2-AmB-LLs to C. albicans hyphae grown *in vitro* is compared in Fig. 1. By measuring the area of red liposome fluorescence from large numbers of epifluorescence images we quantified the binding data for each liposomal type. Both Dectin-1- and Dectin-2-targeted liposomes bound at least 100-fold more efficiently than our pegylated analog of AmBisome, AmB-LLs (Fig. 1D and *P*  =  4.0 × 10^−6^ and 4.5 × 10^−6^, respectively). Bovine Serum Albumin coated liposomes, BSA-AmB-LLs, also did not bind at significant levels. The binding efficiency of the two different Dectin targeted liposomes was not statistically distinguishable (*P*  =  0.18). However, their binding patterns differed. DEC1-AmB-LLs appeared to target exopolysaccharide distally associated with hyphae (Fig. 1A), while DEC2-AmB-LLs appeared to bind exopolysaccharide more proximally associated with hyphae and more evenly distributed throughout colonies of filamentous cells (Fig. 1B). We presume this difference in localization reflects the differential distribution or accessibility of the targets of Dectin-1 and Dectin-2 in the exopolysaccharide matrix.

### A neutropenic mouse model of disseminated candidiasis.

We employed a neutropenic mouse model of immunosuppression to ensure that reproducible and sustainable invasive C. albicans infections were established in all mice (36–39). Neutropenic mice were infected by the intravenous injection of C. albicans yeast cells on day 0 (D0) and subsequently treated with an intravenous injection of DEC1-AmB-LLs or DEC2-AmB-LLs, AmB-LLs, or liposome dilution buffer at indicated times postinfection (PI). The regimens for immunosuppression, infection, treatment, and assays are diagrammed in Fig. S1. The efficacy of targeted and untargeted liposomes or the control buffer was quantified by measuring the association of liposomes with C. albicans infection sites in kidneys, the fungal burden in kidneys, and mouse survival.

### DectiSomes associate with C. albicans infection sites in the kidneys.

**FIG 2:**
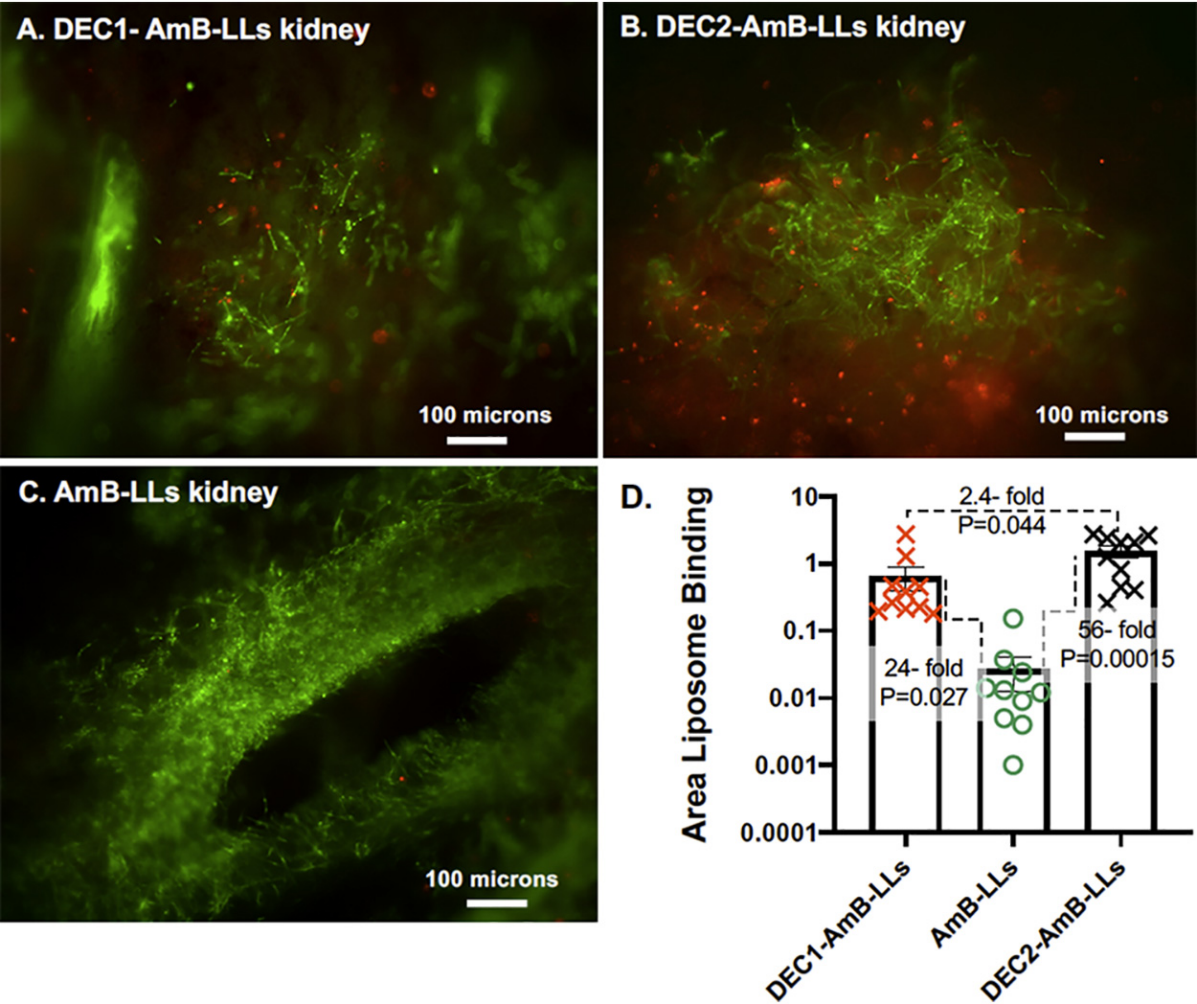
DectiSomes delivered intravenously are concentrated in C. albicans infection centers in the mouse kidney. Immunosuppressed mice with invasive candidiasis were injected intravenously with red fluorescent liposomes. Thick sections of the kidneys were stained with CW. (A, B, C) The blue fluorescence of chitin (shown in green) and the red fluorescence of rhodamine tagged liposomes were photographed by epifluorescence from the surface of the tissue sections at ×10 magnification. (A) AmB-LLs. (B) DEC1-AmB-LLs. (C) DEC2-AmB-LLs. (D) A scatter bar plot compares the area of red liposome fluorescence quantified from 10 images of infection centers for each treatment. Fold differences in the average area of liposome staining and *P* values are indicated.

Here we decided to test if DEC1-AmB-LLs and DEC2-AmB-LLs were preferentially associated with C. albicans cells in infected kidneys compared with untargeted AmB-LLs. Neutropenic mice were intravenously infected with 7.5 × 10^6^ C. albicans yeast cells on D0 and then given two subsequent intravenous doses of rhodamine B tagged targeted DEC1-AmB-LLs, DEC2-AmB-LLs, or untargeted AmB-LLs delivering 0.4 mg/kg AmB 3 h and 24 h PI (Fig. S1A). This amounted to 0.83 mg/kg Dectin protein per treatment for each class of DectiSomes. On day 3 (72 h PI), kidneys were harvested and fresh tissue was hand sectioned. Fungal chitin was stained with calcofluor white (CW) to identify infection sites and the surface of the tissue was examined top down by epifluorescence. The majority of kidney sections contained a few to a dozen infection sites of approximately 100 to 400 microns in diameter (Fig. 2). The rhodamine red fluorescence of DEC1-AmB-LLs, DEC2-AmB-LLs, and AmB-LLs was detected in association with C. albicans hyphae in approximately 20%, 80%, and 5% of the infection sites, respectively (Fig. 2A to C), albeit, the amount of AmB-LLs observed were often at the limit of our detection. We quantified the red fluorescent area of liposome binding within and surrounding infection sites in images wherein liposomes were detected. A scatter bar plot (Fig. 2D) shows that, respectively, DEC1-AmB-LLs and DEC2-AmB-LLs were 24-fold (*P*  =  0.027) and 56-fold (*P*  =  0.00015) more strongly associated with infection sites than AmB-LLs. This analysis gives only a semiquantitative assessment of binding because it does not account for the differing frequency of finding infection sites with the three different types of liposomes. Replicate images of liposome binding are shown in Fig. S2.

### DectiSomes targeting of AmB enhanced the reduction of fungal burden in kidneys.

**FIG 3:**
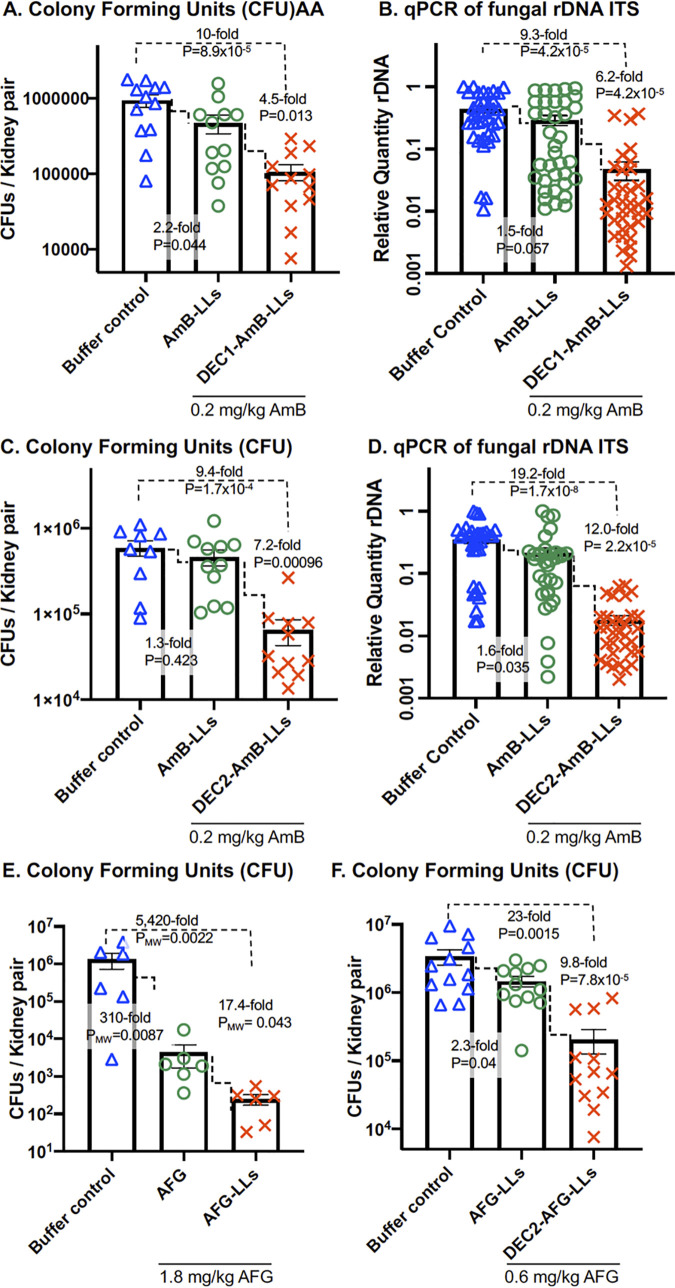
DectiSomes were more effective at reducing the burden of C. albicans in the kidneys compared with untargeted AmB-LLs. Neutropenic mice infected with C. albicans were treated with PBS (buffer control) or the indicated AmB loaded liposomes. Scatter bar plots compare the fungal burden in the kidneys following treatment. (A, B) DEC1-AmB-LLs or AmB-LLs delivering 0.2 mg AmB/kg or PBS. (C, D) DEC2-AmB-LLs or AmB-LLs delivering 0.2 mg AmB/kg or PBS. (E, F) The performance of targeted Anidulafungin loaded liposomes. (E) Comparison of free AFG to AFG-LLs delivering 1.8 mg/kg AFG. (F) Comparison of AFG-LLs to DEC2-AmB-LLs delivering 0.6 mg/kg AFG. CFU or the relative quantity (RQ) of C. albicans rDNA was determined in kidney homogenates from the same mice. Six to 12 mice were included in each treatment group. See treatment regimens displayed in Fig. S1A. Due to the nonparametric distribution of some CFU data *P* values (P_MW_) were calculated using the Mann-Whitney test in Prism.

Using this same regimen of immunosuppression and infection, mice were treated once at 3 h PI with either AmB-LLs, DEC1-AmB-LLs, or DEC2-AmB-LLs delivering 0.2 mg/kg AmB diluted into phosphate-buffered saline (PBS) or with the same amount of PBS alone (buffer control). On day 1, the mice were euthanized and their kidneys excised, homogenized, and assayed for fungal burden. In various previous reports on neutropenic mouse models of candidiasis infected with 10^6^ C. albicans or 10^7^ C. glabrata cells, a single dose of 1.0 to 20 mg/kg AmB delivered intravenously a few hours PI as micellar AmB-DOC or L-AmB produced 3- to 10,000-fold reductions in the kidney fungal burden relative to control mice (38–40). In our mouse model, AmB-LLs delivering 0.2 mg/kg AmB provided only marginal often insignificant reductions in fungal burden relative to PBS treated mice (*P*  =  0.035 to 0.44, Fig. 3). However, mice treated with DEC1-AmB-LLs delivering 0.2 mg/kg AmB showed a 4.5-fold reduction in CFU relative to AmB-LL treated mice (*P*  =  0.013, Fig. 3A). Assays of the relative quantity (RQ) of C. albicans rDNA ITS gene copies on DNA prepared from parallel samples of homogenized kidney tissue from the same mice (Fig. 3B) revealed DEC1-AmB-LL treated mice had a 6.2-fold greater reduction in fungal burden in the lungs than AmB-LL treated mice (*P* = 4.2 × 10^−5^), supporting the CFU results.

Mice treated with DEC2-AmB-LLs delivering 0.2 mg/kg AmB showed a 7.2-fold reduction in the kidney fungal burden relative to AmB-LLs based on CFU (*P* = 9.6 × 10^−4^, Fig. 3C) and a 12-fold reduction based on qPCR amplified rDNA ITS (*P* = 2.2 × 10^−5^, Fig. 3D).

### DectiSomes targeting AFG enhanced the reduction of fungal burden.

AFG is a first-line antifungal used to treat candidiasis, with daily patient doses of 1 to 4 mg/kg continued for several weeks (41, 42). We wished to determine if Dectin targeting of liposomal AFG might improve drug performance. We employed Dectin-2 targeting to test this idea. In published studies using neutropenic mouse models of candidiasis, a dose of 5 to 20 mg/kg of free AFG produces approximately a 10-fold drop in fungal burden in the kidneys within 24 h to 48 h (37, 43). A recent study prepared AFG loaded un-pegylated liposomes (44) and examined them in a wax moth model of candidiasis. The prophylactic administration of AFG-LLs delivering 2.6 mg/kg AFG significantly improved insect survival relative to an equivalent prophylactic dose of free AFG (44). We prepared pegylated AFG-LLs with the same lipid composition as our AmB-LLs (Table S1) and examined their performance in this mouse candidiasis model. Mice were given one intravenous dose of free AFG or AFG-LLs at 3 h PI delivering 1.8 mg/kg of the antifungal. At day 1, free AFG reduced the fungal burden 310-fold relative to a buffer treated control (Fig. 3E, P_MW_ = 0.0087). AFG-LLs reduced the kidney fungal burden an additional 17-fold more than free AFG (Fig. 3E, P_MW_ = 0.043). Then we tested AFG-LLs with or without coating with Dectin-2. One intravenous dose of DEC2-AFG-LLs delivering 0.6 mg/kg AFG reduced the kidney fungal burden 9.8-fold more than untargeted AFG-LLs based on CFU (Fig. 3F and *P* = 7.8 × 10^−5^). This result was supported by qPCR analysis of rDNA levels (Fig. S3).

### Pegylated AmB-LLs outperformed unpegylated AmBisome and targeting by Dectins further improved their performance.

**FIG 4:**
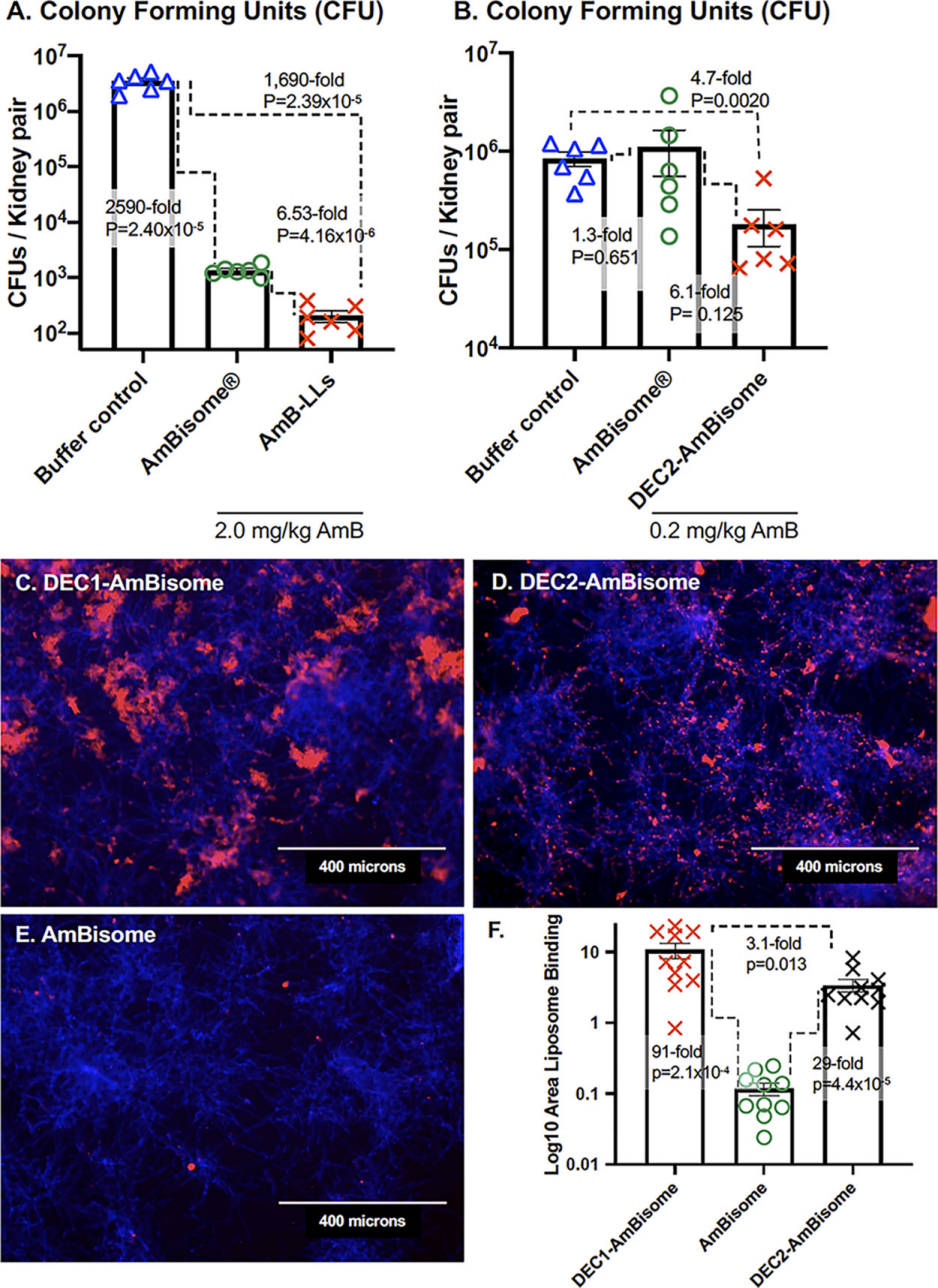
AmBisome, DEC2-Ambisome, and pegylated AmB-LLs compared in fungal burden and fungal binding assays. (A, B) The kidney fungal burden was examined after neutropenic mice infected with C. albicans were treated with liposomes. (A) Mice were treated with AmBisome or AmB-LLs delivering 2.0 mg AmB/kg or PBS. (B) Mice were treated with AmBisome and DEC2-AmBisome delivering 0.2 mg AmB/kg or PBS. See mouse treatment regimens displayed in Fig. S1A. (C, D, E) Fluorescent images showing the binding of Dectin targeted and untargeted rhodamine B tagged AmBisome to *in vitro* grown C. albicans. Fungal chitin was stained with CW. (F) Quantification of the liposome binding was estimated from multiple images such as those in C–E. Standard errors, fold differences in the average area of liposome staining, and *P* values are indicated.

We wished to compare the performance of unpegylated commercial AmBisome to our pegylated AmB-LLs and also to determine to what extent Dectin targeting improved the performance of AmBisome. We found that AmB-LLs delivering 2 mg/kg AmB reduced fungal burden in the kidneys 6.5-fold more than AmBisome delivering the same amount of AmB (*P*  =  4.2 × 10^−6^, Fig. 4A). We prepared Dectin-1 and Dectin-2 coated AmBisome. Dectin-1 and Dectin-2 targeted AmBisome bound to C. albicans hyphae with similar specificity (Fig. 4C to E) and efficiency (Fig. 4F) as Dectin targeted AmB-LLs (Fig. 1). When delivering 0.2 mg/kg AmB, DEC2-AmBisome reduced the kidney fungal burden 6.1-fold more than untargeted AmBisome (*P*  =  0.0125, Fig. 4B).

### DectiSomes increased mouse survival.

**FIG 5:**
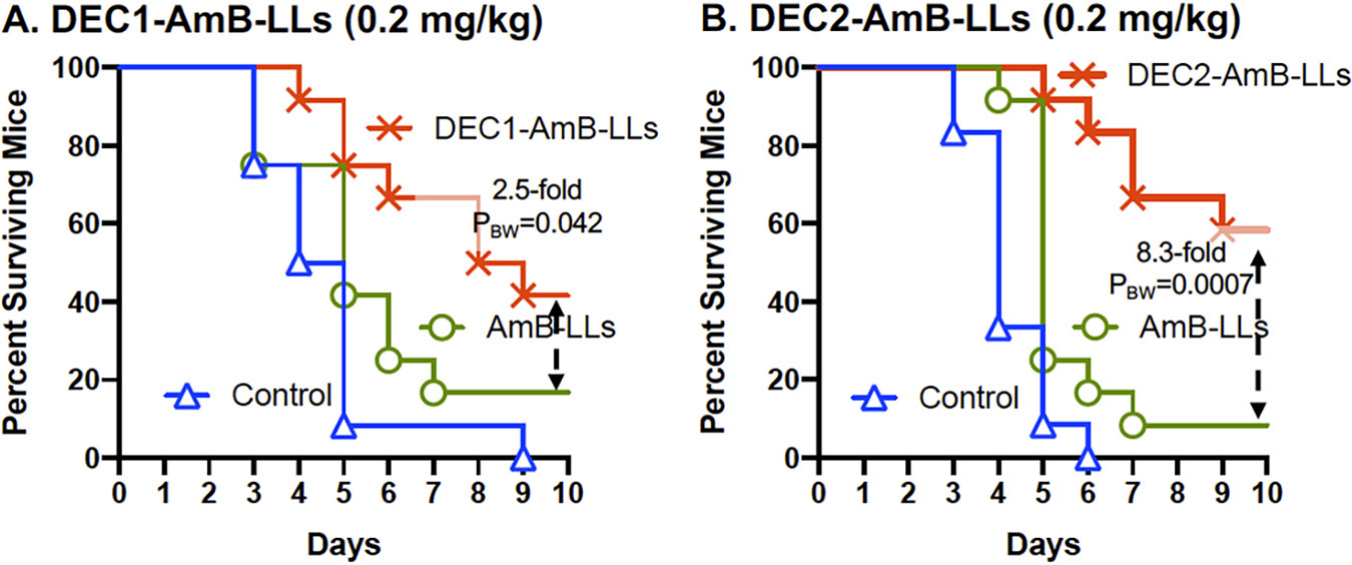
DectiSomes improved mouse survival relative to untargeted AmB-LLs. Neutropenic mice infected with C. albicans were treated with DEC1-AmB-LLs (A), DEC2-AmB-LLs (B) and AmB-LLs delivering 0.2 mg AmB/kg diluted into PBS or with PBS (control). Mouse survival was monitored for 10 days PI. Twelve mice were included in each treatment group. See treatment regimen displayed in Fig. S1B. Fold differences in the percent of surviving mice are indicated. *P* values comparing the survival curves for DectiSome and AmB-LL treated mice were estimated using the Gehan-Breslow-Wilcoxon test (P_BW_) in Prism.

Neutropenic mice were given an intravenous inoculum of 0.5 × 10^6^ C. albicans yeast cells, three intravenous treatments with DEC1-AmB-LLs, DEC2-AmB-LL, or AmB-LLs delivering 0.2 mg AmB/kg or buffer (control) at 3 h PI (day 0), 24 h PI (day 1), and 48 h PI (day 2) (Fig. S1B). Survival was monitored for 10 days PI (day 10) as shown in Fig. 5 (36, 45, 46). All buffer-treated control mice and a few of the liposome treated mice showed reduced grooming by day 3 PI. Fig. 5A presents a survival curve comparing DEC1-AmB-LLs to AmB-LLs. Forty-two percent of the DEC1-AmB-LL treated mice survived to day 10 compared with 16.6% of the AmB-LL treated mice, a 2.5-fold difference in the percent survival. Control mice had an average survival time of 4.6 days. DEC1-AmB-LL treatment increased the average survival time to 8.0 days compared with 5.7 days for AmB-LL treated mice (*P*  =  0.035). Fig. 5B examines the survival of mice treated with DEC2-AmB-LLs. Sixty-six percent of the DEC2-AmB-LL mice survived to day 10 compared with 8.3% of the AmB-LL mice, an 8.3-fold difference in the % survival. Control mice had an average survival time of 4.2 days, AmB-LL mice 5.6 days, and DEC2-AmB-LL mice 8.7 days, based on estimating survival time to day 10. DEC2-AmB-LL treatment significantly increased the average days of survival relative to AmB-LL treatment (P_BW_ = 0.0006). In summary, when mice with invasive candidiasis are treated with Dectin-1 or Dectin-2 targeted DectiSomes delivering 0.2 mg/kg AmB, they both showed significantly improved mouse survival relative to AmB-LL treatment. Dectin-2 appeared superior to Dectin-1 in targeting liposomal AmB.

## DISCUSSION

We showed that the pegylated AmB-LLs and AFG-LLs employed herein outperformed commercial unpegylated AmBisome and free AFG, respectively, at reducing the burden of C. albicans cells in the kidneys. Our AmB-LL and AFG-LLs are pegylated stealth liposomes. Pegylation protects liposomes from opsonization and phagocytosis, which significantly extends the half-life of packaged drug (47–50). Five moles percent of the liposomal lipids in the membrane of our liposomes are the lipid, DSPE (distearoyl-sn-glycero-3-phosphoethanolamine) coupled to poly-ethylene glycol (PEG). DSPE inserts in the liposome membrane and presents PEG on the liposome surface (32). AmBisome was first patented and introduced to the clinic before the benefits of pegylation were thoroughly described. Because of their pegylation, we anticipated that our AmB-LLs might outperform AmBisome. However, while they share 11 moles percent, AmB, AmB-LLs, and AmBisome also have different ratios of different anionic lipids and cholesterol. Hence, because of these compositional differences, we cannot conclude unambiguously that pegylation is the major reason that we observed superior antifungal activity of AmB-LLs over AmBisome. Yet, our results support this concept.

DEC1-AmB-LLs and DEC2-AmB-LLs both bound to the exopolysaccharide matrix associated with *in vitro* grown C. albicans hyphae and their binding efficiency was indistinguishable. Yet, qualitatively the location of their binding in the matrix was distinct, with DEC2-AmB-LLs binding to exopolysaccharide that was more closely and uniformly associated with hyphae than DEC1-AmB-LLs. DectiSomes delivered intravenously efficiently penetrated into the kidneys of infected mice. Modest amounts of DEC1-AmB-LLs and DEC2-AmB-LLs were observed in association with infection sites, while AmB-LLs were barely detected. These data suggest that once they are bound to their glycan ligands, DectiSomes remained in place, but being unbound AmB-LLs must have been flushed out of the kidneys. However, there was a wide variation in the amount of both DectiSomes measured in individual infection sites as revealed by the wide spread of data in scatter bar plots. DectiSomes may not penetrate equivalently into all parts of the infected kidney or the glycan ligands for binding may not be equivalently expressed in all infection sites. Both types of DectiSomes appeared to be associated with hyphae, but not bound directly to them, consistent with their binding to oligoglucans and oligomannans in the associated fungal exopolysaccharide. DEC2-AmB-LLs bound significantly more efficiently to infection sites than DEC1-AmB-LLs. These results suggested the possibility that Dectin-2 targeted AmB-loaded liposomes would likely out-perform Dectin-1 targeted liposomes when their antifungal activity was tested in this mouse model of candidiasis.

Dectin-1 and Dectin-2 targeted DectiSomes delivering 0.2 mg/kg provided approximately a 9- to 19-fold reductions in kidney fungal burden relative to the buffer control and a 5- to 12-fold reduction relative to AmB-LL treated mice. DEC2-AmB-LLs appeared to be slightly superior to DEC1-AmB-LLs at reducing fungal burden relative to AmB-LLs. In mouse survival studies both Dectin-1 and Dectin-2 targeted DectiSomes delivering 0.2 mg/kg AmB improved mouse survival relative to untargeted AmB-LLs. Again DEC2-AmB-LLs appeared superior to DEC1-AmB-LLs, wherein DEC2-AmB-LLs showed relative increases in the average days of survival and the percent of surviving mice.

The majority of these experiments used low final AmB concentrations of 0.2 mg/kg, which meant each mouse received an injection of 100 μl of a 100-fold dilution of our targeted and untargeted liposome preparations (31, 32). In the future we will need to determine the concentrations at which Dectin-1 and Dectin-2 targeted antifungal liposomes completely clear infections and insure mouse survival. We also need to explore their utility against multiple C. albicans isolates and related species including C. glabrata and C. auris. We do not anticipate any additional dose-dependent toxicity issues for Dectin targeted liposomes, because we have previously shown that their toxicity is similar to that of untargeted liposomes when tested against cultured human cells (31, 32).

Dectin-2 targeting of liposomal AFG (i.e., DEC2-AFG-LLs) provided a several fold increase in antifungal efficacy over untargeted AFG-LLs at reducing fungal burden. Targeting allowed a single low dose of AFG, 0.6 mg/kg, to be highly effective, a dose at which untargeted liposomal AFG-LLs was not effective. Although both AmB and AFG are amphiphobic, allowing them to be intercalated into liposomal membranes, the two drugs are distinct in structure and antifungal mechanism. The polyene AmB is generally thought to damage the fungal membrane and osmotic integrity by binding to ergosterol, while the echinocandin AFG inhibits beta-glucan synthase and ultimately weakens the fungal cell wall and exopolysaccharide matrix. Showing improved efficacy for targeted AFG is important step forward, because it begins to generalize DectiSome targeting strategies, paving the way to improve the performance of a wide variety of other existing polyene and echinocandin drugs, other classes of antifungal drugs such as the azoles and antimetabolites, and yet to be clinically approved new drugs. Furthermore, Dectin-2 targeting improved the effectiveness AmBisome, suggesting that DectiSome targeting should improve the antimicrobial activity of nanoparticles with diverse chemical compositions.

Dectin-1 and Dectin-2 are both C-type lectin pathogen receptors that respond to infections by *Candida* spp. and signal the immune system of an ongoing infection. Dectin-1 is expressed primarily by neutrophils, macrophages, and dendritic cells, while Dectin-2 is primarily expressed by dendritic cells. Dectin-2 appears to be the primary receptor by which bone marrow-derived dendritic cells (BMDC) signal an oligomannan dependent innate immune response to C. albicans yeast cells (51). BMDCs from *Clec4n*-*/Clec4n*- (Dectin-2 KO) mice show a 100-fold reduction in the induction of inflammatory cytokines, such as IL-6 or TNF-α, when exposed to C. albicans cell-derived mannans, compared with WT BMDCs (51). The importance and role of Dectin-1 in the response to exposure to *Candida* spp. is less clear, and appears to be less significant. There is substantial evidence that the *Candida* spp. beta-glucan ligands are heavily masked from binding either by Dectin-1 and/or anti-beta-glucan antibodies and significantly protected from the host innate immune response (52–56). For example, when BDMCs derived from *Clec7a*-*/Clec7a*- mice (Dectin-1 knockout [KO]) are exposed to C. albicans or other *Candida* spp. yeast cells, their induction of inflammatory cytokines, such as IL-6 or TNF-α, is only reduced by 20% to 50% relative to wild type BMDCs (57). Yet, Dectin-1 KO mice infected with C. albicans are significantly more likely to die than WT mice, suggesting Dectin-1 does contribute positively to preventing infection. By contrast, the survival of these Dectin-1 KO mice is not reduced, when exposed to other common pathogenic *Candida* spp. such as C. glabrata or C. tropicalis, presumably due to the masking of their oligoglucans (57).

Therefore, based on the response of these two Dectins to *Candida* spp. infections and the suggestion that oligoglucans might be masked during infection, we were confident at the start of this project about the potential of Dectin-2 targeting, but doubtful about the benefits of Dectin-1 targeting. We were encouraged to proceed with *in vivo* testing of Dectin-1 targeted DectiSomes by our strong *in vitro* binding data and modest *in vivo* kidney data showing DEC1-AmB-LL bound to exopolysaccharide associated with C. albicans. We are satisfied with the finding that both DEC1-AmB-LLs and DEC2-AmB-LLs are highly effective in animal models. The slightly lower effectiveness of DEC1-AmB-LLs at reducing fungal burden and improving mouse survival compared with DEC2-AmB-LLs is likely due to partial masking of oligoglucans *in vivo* and the more distal association of DEC1-AmB-LLs with hyphae.

*Canada* spp. form biofilms, which sequester antifungal agents and physically block access to fungal cell surfaces, thus helping them evade the host immune system and increase antifungal drug resistance (30, 58, 59). Even immunocompetent individuals may have persistent *Candida* infections, when biofilms form on implanted medical devices (58, 60, 61). Liposomal Amphotericin B (L-AmB), even unpegylated, is significantly more effective at killing C. albicans residing in biofilms than either detergent solubilized micellar AmB-DOC or micellar fluconazole (62). L-AMB also penetrates more efficiently into various organs (63–65) and the fungal cell wall (66), and show reduced organ toxicity and less infusion toxicity at higher AmB doses when compared to detergent solubilized AmB (26–28, 67, 68). Because of the effectiveness of liposomal formulations at both organ and biofilm penetration, new studies on therapeutics to treat candidiasis, often include L-AMB (e.g., AmBisome) as a standard for comparison (18, 45, 69–71), as we have done herein. If Dectin targeted liposomes penetrate *Candida* biofilms and are able to bind their oligoglycan targets, they should improve antifungal drug performance. Future studies need to focus specifically on the efficacy of DectiSomes against various Candida spp. biofilms.

## MATERIALS AND METHODS

### Strains and culture.

C. albicans strain SKY43 (72) and was derived from a human isolate (SC5314, ATCC MYA-2876) which was subsequently deleted for URA3 (strain CA14, Δura3::imm434/Δura3::434) (73). C. albicans yeast cells were grown to early log phase in YPD, washed once into fresh YPD, aliquoted, snap-frozen in liquid nitrogen, and stored frozen at −80°C in 25% glycerol. Cells were thawed once or twice just before use, vortexed, and diluted to the desired cell concentration in sterile saline. The viability of the thawed cultures was close to 99%. Mice were infected via the retroorbital injection of 100 μl of saline containing 7.0 × 10^6^ or 0.5 × 10^6^ yeast cells (71) (Fig. S1).

Seven- to 8-week-old outbred female CD1 (CD-1 IGS) Swiss mice (27 g to 30 g ea.) were obtained from Charles River Labs. Mice were maintained in UGA’s Animal Care Facility. All mouse protocols met guidelines for the ethical treatment of non-human animals outlined by the U.S. Federal government (74) and UGA’s Institutional Animal Care and Use Committee (AUP #A2019 08–031-A1).

### *In vitro* binding studies.

For *in vitro* binding studies, 10,000 cells/ml C. albicans yeast cells were plated in 500 μl of RPMI 1640 media lacking red dye at pH 7.5 in each well of a 24-well microtiter plate and grown for 12 h to achieve approximately 50% coverage with hyphae. Cells were washed once with PBS, fixed in 4% formalin for 45 min, and washed 3× with PBS. Cells were blocked with PBS + 5.0% BSA for 30 min, treated with rhodamine red fluorescent liposomes in this blocking buffer, stained with 25 uM CW (Blankophor BBH SV-2560; Bayer, Corp.) for 60 min, and washed 3× with the same buffer. Images were taken on an EVOS imaging system using the DAPI and RFP fluorescent channels and the red fluorescence area within un-enhanced images was quantified in ImageJ (31). The accompanying images presented were enhanced equivalently in the blue and red channels.

### Neutropenic model of disseminated candidiasis.

Immunosuppressed neutropenic mice were obtained by treatment with both the antimetabolite cyclophosphamide (CP, Cayman #13849) and the synthetic steroid triamcinolone (TC, Millipore Sigma # T6376) following the schedules shown in Fig. S1. Five or six mice were in each treatment group and in some cases two replicate experiments. CP and TC stocks, dilutions, and injection methods were described recently (35).

Infected buffer control animals not receiving antifungal therapy first showed a ruffled coat due to reduced grooming, then decreased movement, followed by abnormal posture, trembling, and severe lethargy. The onset of symptoms occurred much more rapidly in animals receiving the larger fungal inoculum size and was reduced in animals receiving liposomal AmB. Once mice showed severe lethargy and were moribund, they were sacrificed by cervical dislocation following anesthesia with isoflurane (Animal Use Protocol, A2019 08–031-A1).

### Liposomes and drugs.

We constructed AmB-LLs, DEC1-AmB-LLs, DEC2-AmB-LLs, and BSA-AmB-LLs as described previously (31, 32). Dectisomes contain 1 mole percent Dectin relative to moles of liposomal lipid. Similar to AmBisome they contain 11 moles percent AmB, but our liposomes also contain two moles percent Rhodamine B-DHPE for visualization.

AFG-LLs were prepared in 273 μl batches using a remote loading method analogous to that which we used to prepare AmB-LLs (31, 32). To quantify AFG loading into liposomes, we determined that AFG had an extinction coefficient (17.4 O.D./mg/ml) at A340 in DMSO, using a dilution series and a Bio-Tek Synergy HT microtiter plate reader (Fig. S4). Ten moles percent AFG (1.7 mg, 1.5 μmoles) relative to moles of liposomal lipid was dissolved in 13 μl DMSO and added to 15 μmoles liposomal lipid (260 μl of 100 nm diameter FormuMax liposomes, F10203, Plain). AFG and liposomes were incubated for 72 h at 37°C with gentle tumbling. The AFG-LLs were spun at room temperature for 2 min at 1,000 × *g* to sediment the remaining insoluble AFG that was not loaded into liposomes and did not remain soluble between liposomes. We had predetermined that the solubility of AFG in our liposome loading buffer (10% sucrose, 20 mM HEPES, pH 7.0–7.5, and 5% DMSO) to be (0.31 mg/273 μl). The AFG precipitate (i.e., that not loaded in liposomes) was then dissolved in DMSO and quantified at A340. By subtraction of the insoluble AFG and the predetermined solubility in loading buffer, we calculated that the AFG-LLs contained 6.2 moles percent AFG. One mole percent Dectin-2 modified with a lipid carrier, DEC2-PEG-DSPE, was then added to the AFG-LLs to make DEC2-AFG-LLs (31, 32). See Table S1. If we started with 20 moles percent AFG (3.4 mg, 3.0 μmoles) in the initial loading of liposomes the final AFG concentration in the AFG-LLs was increased to 11 moles percent.

### Liposome binding to infection centers in the kidneys.

Hand sections of freshly harvested kidney were cut and stained for fungal chitin with calcofluor white (Blankophor BBH SV-2560; Bayer, Corp.) as previously described (35). The blue fluorescent chitin ex360/em470 and rhodamine tagged liposomes ex560/em645 were examined by epifluorescence microscopy using a LEICA DM6000 compound fluorescence microscope at ×10 magnification as described previously for liposome binding to lung tissue (35). The area of red fluorescent liposome binding in the original TIFF images was quantified in ImageJ as described previously (35). For photographic presentation of binding, the blue CW and red liposome channels of the original Tiff images were equivalently enhanced in Photoshop (version 20.0.8) to aid in visualizing fungal cells and liposomes, respectively, and then converted to JPEG images for presentation.

### Fungal burden estimates.

Fungal burden was estimated in excised kidney pairs from infected animals on day 1 PI by assaying both the number of CFU and the amount of C. albicans ribosomal rDNA intergenic transcribed spacer (ITS) estimated by quantitative real-time PCR (qPCR). Kidney pairs were weighed and minced into hundreds of approximately 1 mm^3^ pieces, the pieces mixed to account for the uneven distribution of infection centers, and aliquoted into 25 mg samples. CFU. 25 mg of the minced kidney tissue was homogenized for 60 s in 200 μl of PBS using a hand-held battery powered homogenizer (Kimble, cat#749540-0000) and blue plastic pestle (Kimble Cat#749521-1500). The homogenate was spread evenly by shaking with sterile glass beads on 5-mm thick YPD (yeast extract, peptone, and dextrose) agar plates containing 100 μg/ml each of Kanamycin and Ampicillin. After a 11-h incubation at 37°C, the microcolonies of 5 to 300 microns in diameter were counted on an EVOS imaging system (AMG Fl) at ×4 magnification. An example of the images of microcolonies used to make CFU estimates is shown in Fig. S5. The number of CFU was corrected for the area of the entire plate relative to each microscopic field (1,289 4× fields/plate) and the weight of each kidney pair. The numbers of colonies were often so low for some of the DEC1-AmB-LL and DEC2-AmB-LL treated mice that many of the 4× fields had zero colonies. In these cases, the plates were grown for a total of 19 h and mature colonies were counted. *qPCR.* DNA was extracted from 25-mg parallel samples from kidney homogenates using Qiagen’s DNeasy blood and tissue kit (#69504) modified as we described previously for A. fumigatus infected lung tissue (35). We typically obtained 25 to 40 μg of total DNA from 25 mg of kidney tissue. Quantitative real-time PCR (qPCR) was used to estimate the amount of C. albicans *rDNA* ITS sequence in 100 ng samples of infected kidney DNA using the conditions described previously (35). Several new PCR primer pairs designed to amplify the ITS downstream of 18S rDNA of C. albicans were designed and tested against purified C. albicans DNA. The optimal primer pair giving the lowest cycle threshold value (Ct) and a single dissociation peak had the following sequences (forward primer Ca18S-4S, 5′-TAGGTGAACCTGCGGAAGGATCATTA and reverse primer Ca18S-2A 5′-TTGTAAGTTTAGACCTCTGGCGGCA). This primer pair gave no detectable product even after 45 cycles of PCR, when uninfected kidney tissue DNA was examined. The RQ of C. albicans *rDNA ITS* was determined by normalizing all Ct values to the lowest Ct value determined for infected control kidneys using the dCt method (75).

### Data management.

Raw quantitative data were managed in Excel v16.16.27. Scatter bar plots, survival plots, and XY plots were prepared in GraphPad Prism v.9.0.0. Because most of the data for liposome binding and fungal burden estimates were reasonably normally distributed, the Student’s two-tailed t-test was used to estimate *P* values (76). Exceptions to estimating *P* values by the *t* test are noted in appropriate figure legends.
