# Supplementary material for: DectiSomes: Glycan Targeting of Liposomal Drugs Improves the Treatment of Disseminated Candidiasis
Source: Antimicrob Agents Chemother. 2022 Jan 18;66(1):e01467-21. doi: 10.1128/AAC.01467-21 (PMC8765427; doi:10.1128/AAC.01467-21)

Supplemental Tables and Figures.

Supplemental Table ST1. Chemical composition of Anidulafungin loaded liposomes (AFG-LLs).

Compound	µg in each preparation of liposomes	µg / µmole Mol. Wgt.	µmoles total	Moles percent relative to moles of liposomal lipid
DSPC (1,2-distearoyl-sn-glycero-3-phosphocholine)	6516.00	790.00	8.25	50.00
cholesterol	1591.00	387.00	4.11	45.00
mPEG2000-DSPE	2302.10	872.00	2.64	5.00
Total mmoles and moles percent liposomal lipid			15.00	100.00
Anidulafungin (AFG)	1030.00	1140.00	0.90	6.02
Lissamine Rhodamine B 1,2-Dihexadecanoyl-sn-Glycero-3-Phosphoethanolamine, Triethylammonium Salt	400.00	1333.81	0.30	2.00

Supplemental Fig. SF1. Treatment regimens used to assay liposome binding, fungal burden and mouse survival- immunosuppression, infection, liposome treatments and endpoints. A neutropenic mouse model was used to ensure reproducible infection by *C. albicans* yeast cells. **A.** The regimen used to assay binding of DectiSomes to infection centers in the kidneys as compared to AmB-LLs and the impact of DectiSomes on fungal burden as compared to untargeted liposomes or control buffer. Liposome binding was assayed on Day 4 (D4) PI and fungal burden was determined on D1 PI. **B.** Regimen used to assay mouse survival after treatment with DEC1-AmB-LLs, DEC2-AmB-LLs, AmB-LLs or control buffer. The Day(s) (D) of treatment are indicated before and after the day of infection (D0). CP, cyclophosphamide. TC, triamcinolone. Survival was monitored until D10.

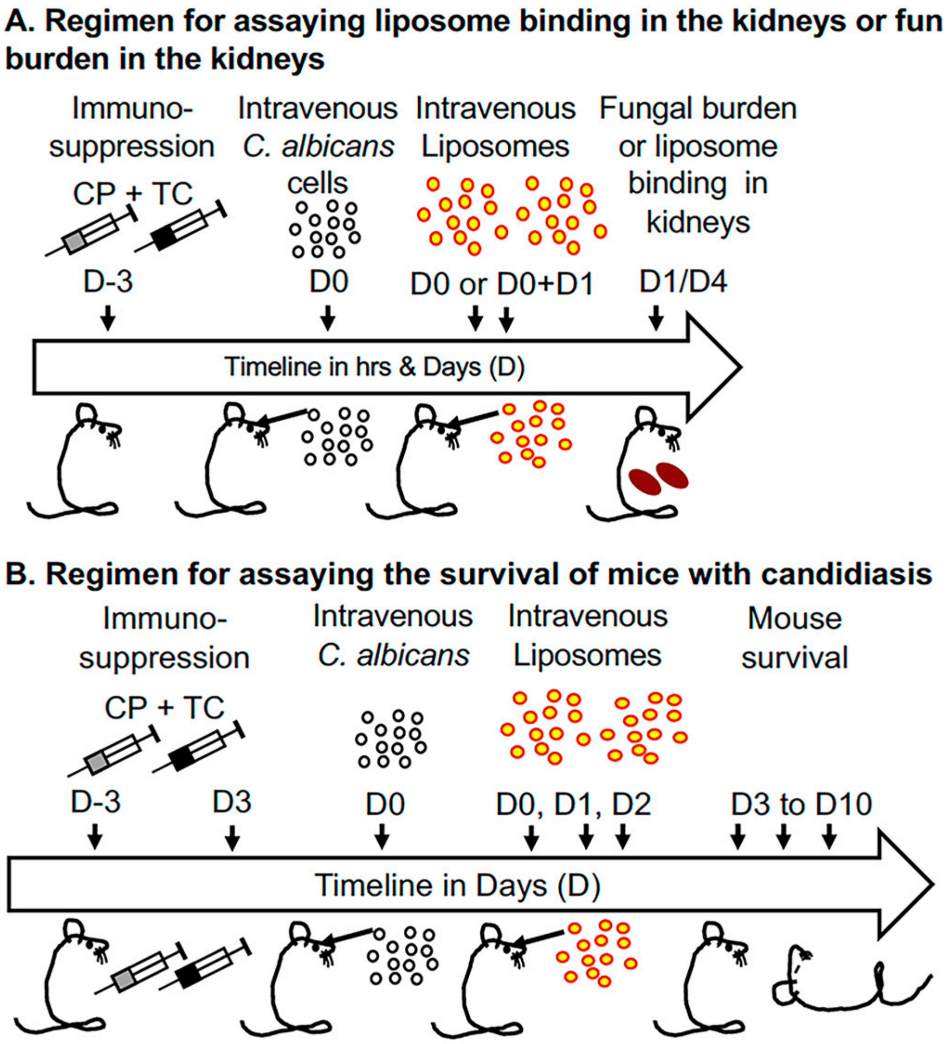

Supplemental Fig. SF2. **DectiSomes targeted by Dectin-1 or Dectin-2 and delivered intravenously are concentrated in *C. albicans* infection centers in the mouse kidney.** Replicate images for the experiment illustrated in **Fig. 2** showing the superior binding of DEC1-AmB-LLs and DEC2-AmB-LLs to *C. albicans* colonies in the kidneys relative to untargeted AmB-LLs.

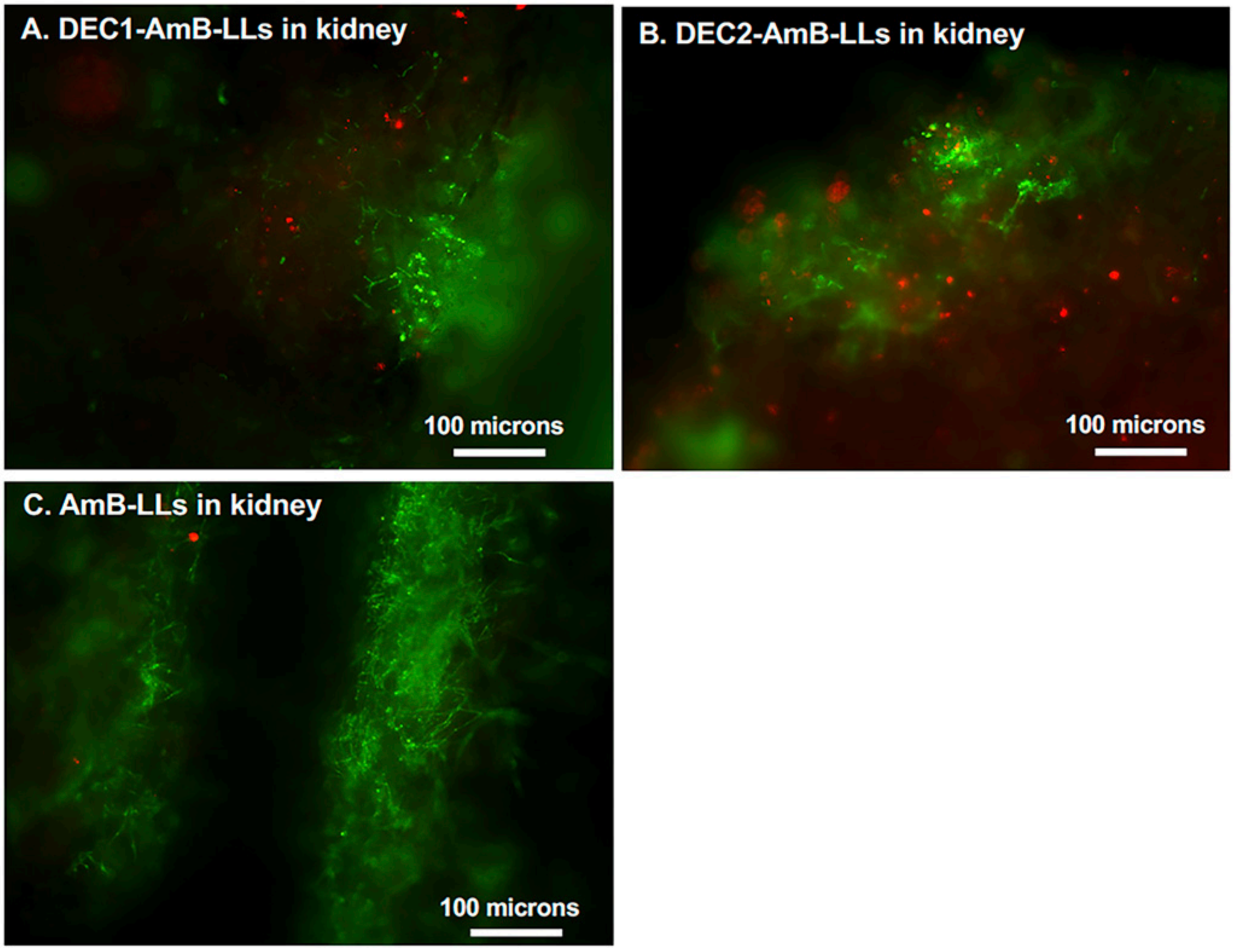

Supplemental Fig. SF3. Dectin-2 coated anidulafungin loaded liposomes, DEC2-AFG-LLs, delivering 0.6 mg/kg AFG were more effective at reducing the burden of *C. albicans* in the kidneys than untargeted AFG-LLs. Relative Quantity (RQ) of *C. albicans* rDNA was determined by qPCR analysis of DNA isolated from duplicate kidney samples to those in **Fig. 3E**, serving as a confirmation of the CFU data**.**

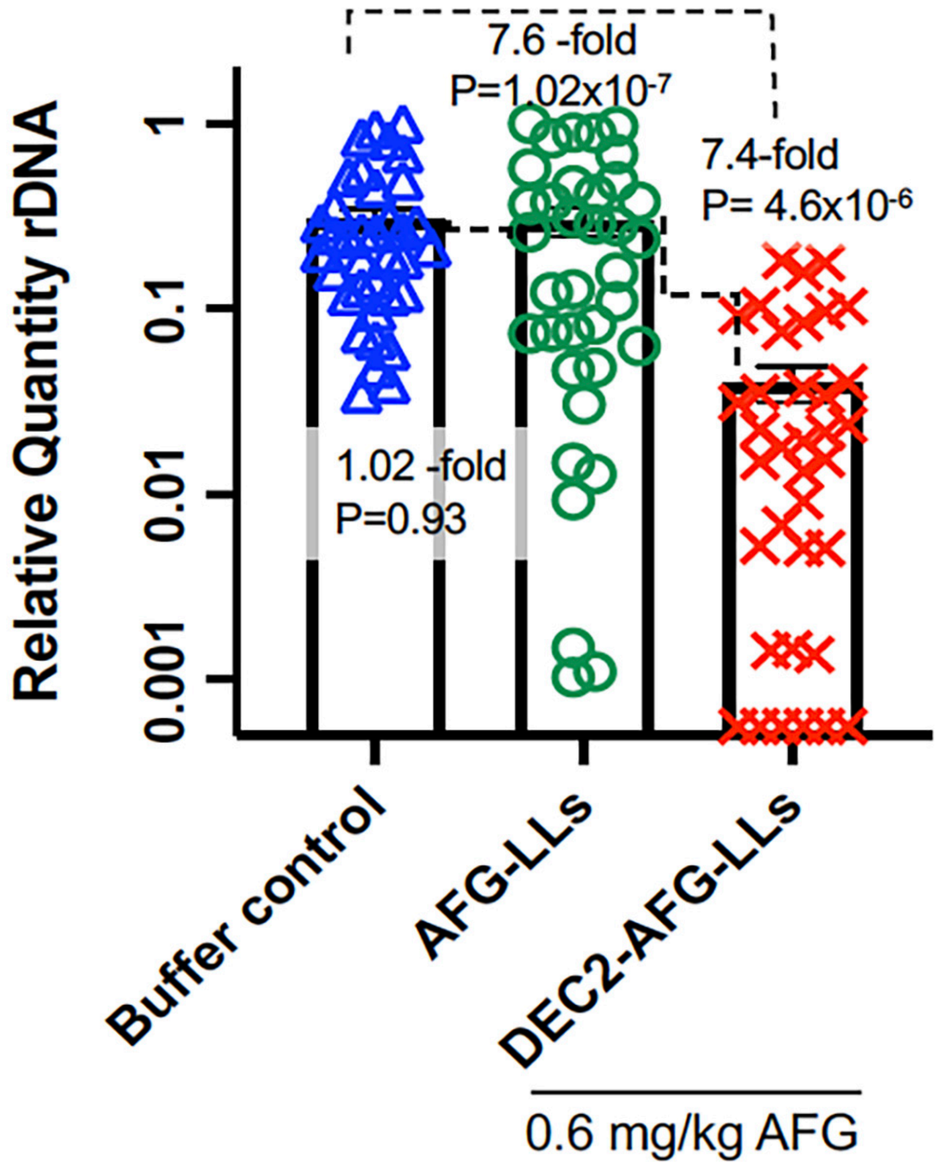

Supplemental Fig. SF4. Experimental data used to quantify the moles percent of AFG loaded into AFG-LLs. Light scattering from liposomes prevents a direct measurement of the UV absorbance of drugs loaded into liposomes. Hence, we estimated the percent of AFG loaded into liposomes by the subtraction of that which was not loaded into liposomes as we have done before to estimate the loading of AmB (Ambati et al., 2019, mSphere 4:1-15). **A.** Standard curve plotting the absorbance of AFG at A340 in a dilution series in a 96 well microtiter plate vs the amount mg amount of AFG dissolved in 50 uL of DMSO. When more than 0.12 mg of AFG were examined the A340 absorbance values were too high to be read. **B.** When 1.7 mg of AFG was tumbled for 3 days in 273 uL of liposome buffer, was 81.3% of the total remained insoluble. This was determined by taking the insoluble AFG precipitate and dissolving it in 50 uL DMSO and reading A340 for a dilution series. **C.** After incubating liposomes and 1.7 mg of AFG together in 273 uL the insoluble AFG was spun down and assayed by in a dilution series in 50 uL DMSO. This is the amount of AFG not taken up by liposomes and not soluble in the buffer surrounding them. **D.** The amount of AFG that remained soluble in 273 uL of liposome buffer was estimated by subtracting the data in curve B from that in A.

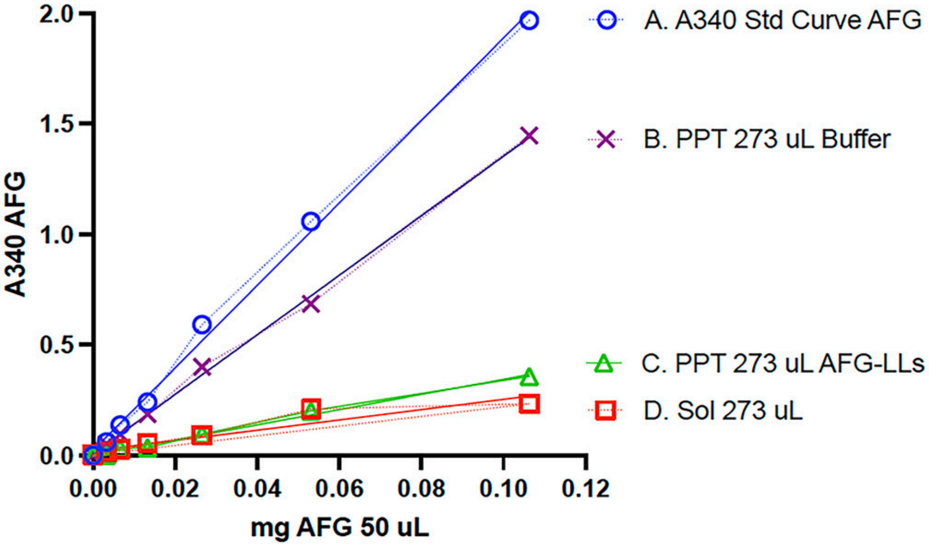

Supplemental Fig. SF5. **Assays of *C. albicans* microcolonies to determine fungal burden in the kidneys of neutropenic mice with candidiasis than AmB-LLs (an example experiment).** Aliquots of homogenized kidney tissue were diluted into PBS, plated on YPD agar, incubated 11 hr at 37°C, and microcolonies counted. **A.** A bar plot compare the average number of CFUs of *C. albicans* for mice treated once with DEC2-AmB-LLs or AmB-LLs delivering 0.2 mg/kg AmB or with liposome dilution buffer. Standard errors are indicated by a line and whisker. **B, C, & D.** Examples of the images used to make CFU estimates. Microcolonies ranging from 5 to 300 microns in diameter were counted from the bottom of agar petri plates on an EVOS imaging system at 4X magnification. The number of CFUs was corrected for the area of the entire plate relative to each microscopic field, the amount of homogenized kidney tissue plated, and the weight of each kidney pair. Six mice were in each treatment group in this example.

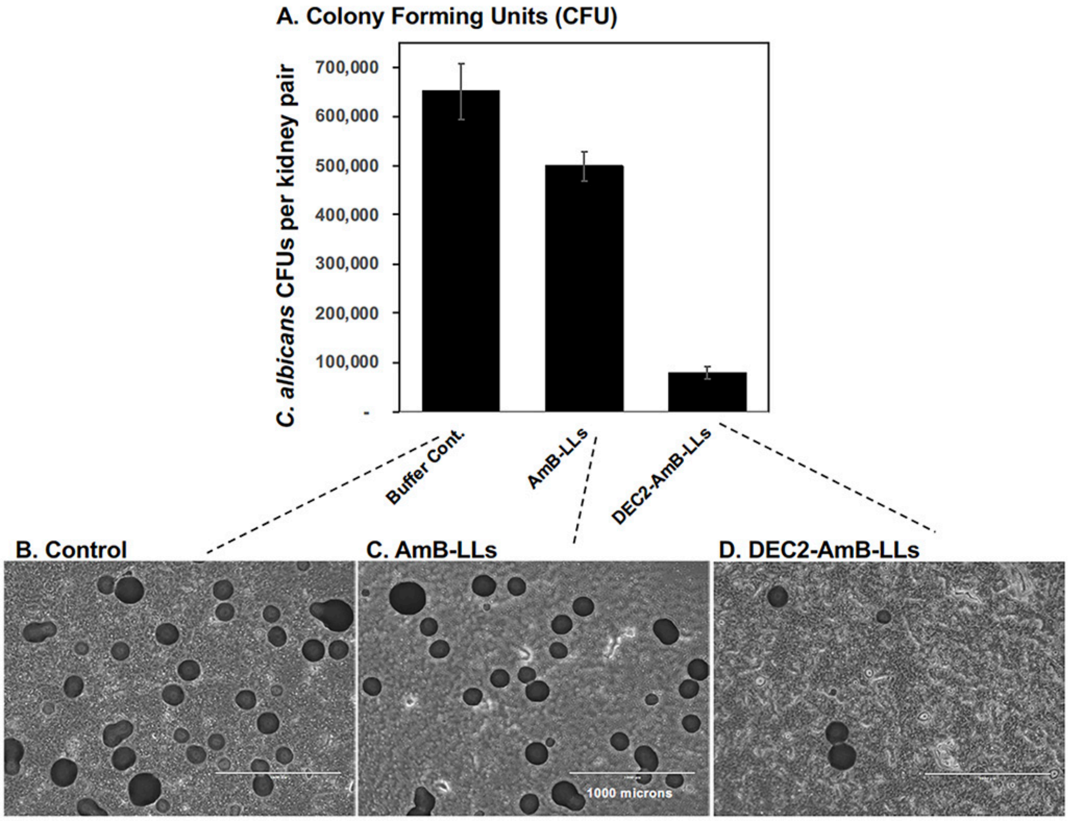

Supplement: Supplemental file 1 — Supplemental material. Download AAC.01467-21-s0001.pdf, PDF file, 1.2 MB [file aac.01467-21-s0001.pdf]
